# Socioeconomic position across the life course and falls among middle- and older-aged adults: protocol for a systematic review

**DOI:** 10.1136/bmjopen-2024-087971

**Published:** 2025-01-21

**Authors:** Frerik Smit, Anita van Zwieten, Catherine Sherrington, Marcia R Franco, Stéphane Cullati, Fiona M Blyth, Saman Khalatbari-Soltani

**Affiliations:** 1 The University of Sydney School of Public Health, Sydney, New South Wales, Australia; 2 Centre for Kidney Research, Children’s Hospital at Westmead, Westmead, New South Wales, Australia; 3 Sydney Musculoskeletal Health, Faculty of Medicine and Health, The University of Sydney, Sydney, New South Wales, Australia; 4 Institute of Musculoskeletal Health, Sydney Local Health District, Sydney, New South Wales, Australia; 5 Population Health Laboratory (#PopHealthLab), University of Fribourg, Fribourg, Switzerland; 6 Quality of Care Service, University Hospitals of Geneva, Geneva, Switzerland; 7 ARC Centre of Excellence in Population Ageing Research (CEPAR), University of Sydney, Sydney, New South Wales, Australia

**Keywords:** Systematic Review, Health Equity, PUBLIC HEALTH

## Abstract

**Abstract:**

**Introduction:**

Individuals experiencing disadvantaged socioeconomic positions (SEPs) may be at increased risk of falls during middle and older age, and these impacts of socioeconomic factors may vary according to the duration, timing and sequencing of exposures across the life course. However, there has not been a recent systematic review of this evidence. This study, therefore, aims to synthesise existing knowledge on the association between SEP across the life course and falls within middle- and older-aged adults.

**Methods and analysis:**

We systematically searched for literature in three academic databases from database inception to 15 March 2024: MEDLINE (Ovid), Embase (Ovid) and PsycInfo (Ovid). The search strategy combined MeSH headings and search terms related to SEP, falls, middle- and older-aged adults and observational studies. Cohort, case-control and cross-sectional studies with mean or median participant age of >40 years, which report on the association between at least one socioeconomic indicator across the life course and one fall outcome and are published in peer-reviewed academic journals were included. No language or geographic restrictions were imposed. Titles and abstracts were screened by one reviewer with 20% of titles and abstracts also screened by a second reviewer. Two reviewers independently screened full texts. Data will be extracted using a standardised Excel template. Using a modified Quality in Prognosis Studies (QUIPS) tool, the risk of bias of included studies will be assessed by one reviewer with 20% of studies also independently appraised by a second reviewer. Meta-analyses will be conducted if sufficient homogeneity between studies permits. Otherwise, a narrative synthesis of results will be undertaken.

**Ethics and dissemination:**

As this is a review of published literature, no ethics approval is required. Findings will be disseminated through a journal article publication, conference presentations and plain-text summaries for public accessibility.

**PROSPERO registration number:**

CRD42024534813.

**STRENGTHS AND LIMITATIONS OF THIS STUDY**

- This review will systematically synthesise existing literature to comprehensively describe associations between socioeconomic position across the life course and falls.
- We will use a comprehensive and systematic search strategy across three key health databases.
- Our review will incorporate considerations of included studies’ risk of overadjustment bias, which is an important form of bias particularly pertinent to reviews of socioeconomic health inequities.
- Insufficient homogeneity between included studies may prevent us from undertaking a quantitative synthesis.

## Introduction

 Falls are defined by the WHO as ‘inadvertently coming to rest on the ground, floor or lower level’.^1^ Falls place a significant burden on the health and well-being of older adults, with the annual incidence of falls among older people being estimated at 26.5% globally^2^ and being projected to increase over time.^3^ The consequences of falls can be serious and include fall-related injuries such as broken bones and head injuries that may require hospitalisation.^4^ In fact, falls represent the second leading cause of death from unintentional injuries worldwide.^5^ Correspondingly, falls can result in disability that limits older adults’ capacity for self-care and participation in activities, thereby impacting their quality of life.^6^ In addition, older adults who have experienced a fall often develop anxiety and fear of falling that further constrains everyday life.^7^ Middle-aged adults also commonly experience falls with significant consequences.^8^ Estimates suggest that, among populations of middle-aged adults, fall rates vary between 8.7% and 31.1% and rapidly increase with age.^9^ Falls among both middle- and older-aged adults are therefore a significant public health issue that needs to be addressed, particularly in the face of our rapidly ageing global population.^10^

Socioeconomic position (SEP) refers to an individual or group’s societal position as determined by an array of social and economic factors including education, occupation, income, wealth and housing.^11 12^ Research has shown that SEP is a highly influential determinant of health among middle- and older-aged adults, as exemplified by the burden of multimorbidity of physical and mental disorders within these age groups being inequitably distributed along socioeconomic dimensions.^13^ Further, the process of healthy ageing itself has been found to be determined by SEP.^14^ Nonetheless, there are current knowledge gaps in our understanding of the extent and pathways to which SEP impacts middle- and older-aged adults’ health. This particularly holds true with regard to SEP throughout the life course. Despite recent studies having taken a life course approach to analysing the impact of SEP on the health of middle- and older-aged adults,^15 16^ research on the long-term impact of SEP at different periods of the life course and SEP trajectories remains limited when it comes to studies focused on functional health outcomes including falls. Given the burden that falls place on the health of adults in middle and older age, it is therefore important to examine whether SEP inequities in falls exist within these age groups and how they manifest in terms of life course exposures to SEP. For example, is there a sensitive period during the developmental stages of one’s life where exposure to (dis)advantaged SEP has a more pronounced impact on the risk of falls in middle and older age? Does persistent exposure to (dis)advantaged SEP result in an accumulating falls risk in middle and older age? Does the sequential pathway between different (dis)advantaged SEP exposures across the life course drive one’s future risk of falls? Or do changes in SEP exposures across the life course determine one’s risk of experiencing a fall as a middle- or older-aged adult?^17^ Gaining this understanding with assistance from these life course conceptual models can subsequently help to identify populations who are at a disproportionate risk of falling, as well as inform the development of effective interventions and policies tackling this public health issue and the periods of the life course at which they should be implemented.

Prior systematic reviews have reviewed the relationship between falls and other socio-demographic variables—namely age, gender, race and ethnicity.^18^,^20^ However, to the best of our knowledge, only one existing systematic review has reviewed the relationship between SEP factors and falls.^21^ This review focused specifically on community-dwelling older adults, and it did not take a life course perspective on analysing the association between SEP and falls. One other systematic review has assessed existing evidence on falls across the life course,^22^ but it did not assess the impact of SEP. Therefore, there is a need for a comprehensive systematic review analysing the body of evidence on the association between SEP and falls across the life course that focuses on both middle- and older-aged adults.

### Objectives

This study aims to systematically review existing literature related to the following research questions:

1. What is the association between SEP across the life course and falls among middle- and older-aged adults?
2. What life course approaches and conceptual models have been used in studies of associations between SEP and falls among middle- and older-aged adults, and what are their corresponding findings?

## Methods and analysis

### Protocol design and registration

The development of this protocol followed the Preferred Reporting Items for Systematic Reviews and Meta-Analyses Protocols statement and checklist, as presented in online supplemental file 1.^23^ It has also been registered in the International Prospective Register of Systematic Reviews (http://www.crd.york.ac.uk/PROSPERO) (CRD42024534813).

### Eligibility criteria

#### Population

The population of interest for this review is middle- and older-aged adults. As such, studies will be restricted to those with a mean age of >40 years or, if the mean is not reported, median age of >40 years. No exclusions will be made based on geographic location, language or date of studies.

#### Exposure

The exposures of interest for this review are SEP indicators at individual-, household-, neighbourhood- and area-levels. Common indicators of SEP include—but are not limited to—education, income, wealth, occupation, employment and housing.^11^ This review will include studies looking at the association between at least one indicator of SEP, at any life course stage, and the outcome of interest.

#### Comparators

Individuals and/or groups in a lower SEP will be compared with individuals and/or groups in a higher SEP, and vice versa.

#### Outcome

The outcome of interest for this review is falls, including the occurrence or rate of falls, injurious and non-injurious falls, fall-related hospitalisations, repeat falls, fall-related injuries and fall-related deaths.

#### Types of studies

This review will include observational studies with cohort, cross-sectional or case-control designs, published in peer-reviewed journals. As our review focuses on an epidemiological/etiological research question, predictive modelling and risk factor studies will only be included if they report unadjusted estimates of the association between a minimum of one indicator of SEP and falls (with results from the full prediction model excluded). Ecological studies, qualitative studies, reviews, commentaries, case series, case studies, dissertations, theses, books, reports, working papers and conference abstracts will be excluded.

#### Search strategy

Searches were conducted in MEDLINE (Ovid), Embase (Ovid) and PsycInfo (Ovid) from inception to 15 March 2024. The searches were conducted using the MeSH headings and search terms outlined in table 1, with intra-category and inter-category terms and/or headings combined using the Boolean operators “OR” and “AND”, respectively.

**Table 1:** Search strategy

Category	MeSH headings and/or search terms
(1) Socioeconomic position	MeSH heading(s):exp Socioeconomic Factors/Search terms:socioeconomic*.tw, socio?economic*.tw, income.tw, wealth.tw, education*.tw, occupation*.tw, employment.tw, hous*.tw, social disparit*.tw, social inequ*.tw, social class.tw, social status.tw, social position.tw
(2) Falls	MeSH heading(s):Accidental Falls/Search terms:fall*.tw, faller*.tw
(3) Middle- and older-aged adults	MeSH heading(s):Exp Aged/, Middle Aged/Search terms:senior*.tw, elder*.tw, old*.tw, aged.tw, ag?ing.tw, middle-age*, middle?age*.tw, mid?life.tw, midlife.tw
(4) Observational studies	MeSH heading(s) (for use in MEDLINE and Embase):Observational Study/, exp Cohort Studies/, exp Case-Control Studies/, Cross-Sectional Studies/Search terms (for use in PsycInfo):observational*.tw, cohort*.tw, case?control*.tw, cross?sectional*.tw, longitudinal*.tw, prospective*.tw, retrospective*.tw

#### Study selection

Search results were uploaded to the COVIDENCE online systematic review software (https://covidence.org/),^24^ which was used to de-duplicate search results. This software was also used for screening titles/abstracts and full texts.

The selection of studies was conducted in two parts. First, the titles and abstracts of 20% of unique search results were screened independently by two reviewers (FS and MRF) to ensure the accuracy and reliability of screening, with differences resolved by discussion. The remaining 80% of titles and abstracts were screened by a single reviewer (FS). Second, full-text retrieval of shortlisted studies was conducted, with all retrieved full texts subsequently being screened independently by two reviewers (FS and MRF). Any disagreements or inconsistencies will be resolved through discussion between the two reviewers (FS and MRF), and consultation with a third reviewer (SK-S or AvZ) where needed.

#### Data extraction

Data extraction will be conducted by one reviewer (FS) into a data extraction form in Microsoft Excel, with all extracted data cross-checked by a second reviewer (MRF). Disagreements will be resolved through inter-reviewer discussions and referral to a third reviewer (SK-S or AvZ) if required. The data items of interest that will be extracted include study and participant characteristics (eg, citation, study design, year of publication, study setting and location, start date and duration, follow-up period, eligibility criteria, sampling method, sample size, participation rates, distribution of participant characteristics); exposure and outcome details (nature and timing of SEP and falls indicator measurements) and study results (direction and magnitude of effect, analytic method and covariates included). Data from studies published in a language other than English will be translated using Google Translate.

**Figure 1:**
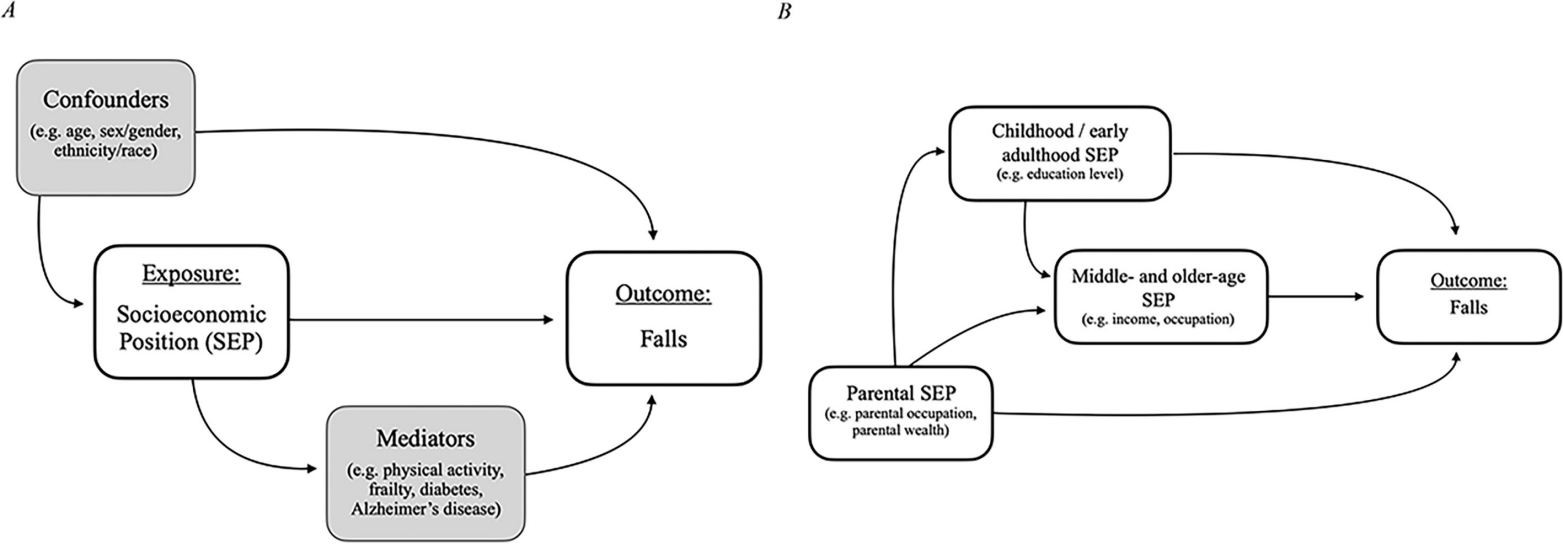
Simplified causal structures for the association between socioeconomic position and falls. (**A**) Socioeconomic position and falls simple causal structures; (**B**) more complex associations between socioeconomic position measured at different life stages across the life course and falls (confounders and mediators are not drawn in (**B**) for simplicity).

Based on our research question, we have developed a simplified causal diagram as depicted in figure 1A to clearly define potential confounders (ie, age, sex/gender, ethnicity/race-related measures) and mediators (eg, health-related behaviours, comorbidities, physical activity) of the association between SEP and falls.^25^ Correspondingly, we have placed restrictions on the types of models and specific estimates that we will extract from different study types, to ensure that we extract data from the most appropriate models and to avoid table 2 fallacy by not interpreting statistical estimates of secondary variables from multivariable models (eg, confounders, effect modifiers).^26^ For studies that specifically aimed to examine the association between SEP and falls, both unadjusted and the most appropriately adjusted models (confounder-adjusted models that do not include mediators) will be extracted. For studies with models of greater complexity (eg, considering the timing and duration of SEP indicators—figure 1B), we will determine the appropriateness of inclusion on a case-by-case basis based on the authors’ stated model assumptions. For predictive modelling and risk factor studies, we will only extract unadjusted models as the estimates from models that include several predictors of interest are not an appropriate reflection of the etiological association between an explanatory variable and an outcome.^27^ This is because these types of studies are focused on developing the most accurate model for predicting an outcome rather than accurately modelling the association between specific exposure variables (eg, SEP) and an outcome, and therefore typically do not consider the role of confounding or mediating variables in their models.^27 28^ As such, it would not be appropriate to etiologically interpret the effect of individual variables from these models.^29^ For all study types, relevant models stratified by age and/or sex will be collected.

Finally, data items will also be collected related specifically to life course approaches undertaken within studies. We will ascertain whether an explicit mention of the life course was made in the study aim(s), method and discussion. If so, we will also extract the specific life course approaches and conceptual models considered by the authors (eg, sensitive period, pathway, accumulation and social mobility).^17^

#### Risk of bias assessment (quality appraisal)

The risk of bias of all included studies will be assessed by one reviewer (FS) using a modified Quality in Prognosis Studies (QUIPS) critical appraisal tool,^30^ with a second reviewer (MRF) also independently assessing a sample of 20% of included studies to ensure the reliability of study quality appraisal. Disagreements will be resolved through discussions between both reviewers (FS and MRF) and consultation with a third reviewer (SK-S or AvZ) where necessary.

While QUIPS was originally designed to assess the risk of bias in prognostic factor studies, it covers all of the principal biases that need to be appraised for etiological studies, namely selection, information, measurement and confounding bias,^31^ as well as statistical analysis and reporting bias. Further, we are using a modified version of the QUIPS tool rather than using Risk of Bias in Non-Randomised Studies of Exposure (ROBINS-E) due to concerns that have been brought up about the latter.^32^ These concerns include that ROBINS-E is challenging to implement feasibly in practice due to its lengthy and time-consuming nature.^32^ QUIPS therefore provides a suitable alternative that is feasible to use and effective in assessing a study’s risk of bias.

We will modify QUIPS by changing the wording of item 3: using ‘exposure measurement’ instead of ‘prognostic factor measurement’. Further, we will also add the following two items related to confounding from the ROBINS-E tool^33^ to assess a study’s risk of overadjustment bias (where variables lying on the causal pathway are adjusted for in estimates of the total effect),^34^ which is a form of bias that is important and has historically been insufficiently considered in systematic reviews of socioeconomic inequities in health:^25^

- Did the authors control for any variables after the start of the exposure period being studied that could have been affected by the exposure?
- Did the authors control for time-varying factors or other variables measured after the start of the exposure window being studied?

#### Data synthesis

Meta-analyses will be conducted for SEP indicators that have adequate numbers of included studies of sufficient homogeneity (eg, study design, measure of exposure and outcome, confounding adjusted). Meta-analyses will be conducted through pooling estimates of effect on the association between SEP and falls using random effects models in R. These models will subsequently produce pooled estimates of effect that will be reported with their corresponding 95% CIs. The extent of publication bias will be assessed through the creation and visual inspection of funnel plot diagrams where there are 10 or more studies and the use of the Egger test.^35^ We will also assess the level of heterogeneity between the studies included in meta-analyses using I^2^ statistics and consider an I^2^ statistic of greater than 50% as indicating significant heterogeneity.^36^ In the event that we observe heterogeneity, we will undertake subgroup and sensitivity analyses to investigate the heterogeneity’s potential explanations. Subgroup analysis will be conducted based on (1) age, (2) sex, (3) ethnicity/race and (4) comorbidities. In addition, sensitivity analysis will be conducted based on (1) study design, (2) study risk of bias (through excluding studies with a high risk of bias) and (3) exposure ascertainment method.

If meta-analyses cannot be conducted, a narrative synthesis by SEP indicator will be conducted. Similarly, if meta-analyses are only possible for certain SEP indicators, a narrative synthesis of the remaining SEP indicators will also be undertaken. Where feasible, visual presentations of our narrative syntheses will be created.

We will also conduct a synthesis of our data from a life course perspective. First, we will narratively synthesise the extent to which existing literature on this topic explicitly mentions and analyses the life course and life course conceptual models, along with corresponding reported results. Second, if sufficient studies contain SEP indicators at different periods across the life course, we will synthesise results through the framework of life course models. More specifically, we will assess whether results support the following models, which have been described in detail elsewhere:^17^ sensitive period, pathway, accumulation and social mobility.

### Patient and public involvement

None.

## Ethics and dissemination

As this is a systematic review of published literature, no ethical approval is required for this study.

Following the completion of the study, a manuscript will be produced for publication in a peer-reviewed academic journal. Our results will be further disseminated through presentations at scientific conferences. Finally, we will summarise our findings in plain text and use social and/or other forms of media to make them accessible to the wider public.

## Discussion

Strengths of this review include the comprehensive search strategy employed across three major academic databases related to health along with explicit consideration of life course processes and overadjustment bias, both of which are important considerations for systematic reviews of socioeconomic health inequities.^17 25^ Our selection criteria and data synthesis methodology will also consider table 2 fallacy and confounding bias by only synthesising the results of the most appropriate models from included studies.^25 26^

One limitation of this study will include the fact that 80% of titles and abstracts are only screened by one reviewer, with just 20% also screened by a second independent reviewer. This increases the risk that relevant studies may be mistakenly excluded. Another potential limitation is the expected lack of homogeneity across studies, which may prevent a quantitative synthesis from being undertaken. Similarly, given that no inclusion restrictions are placed based on study language, geography, date and setting, considerable heterogeneity across study contexts is expected. Therefore, meta-analyses or subgroup analyses may not be feasible. However, we will discuss this heterogeneity in detail through narrative synthesis.

To the best of our knowledge, our study will be the first comprehensive systematic review to synthesise existing knowledge on the association between SEP and falls among middle- and older-aged adults through a life course perspective. Our findings may help to inform interventions and policies aimed at reducing falls and fall inequities within these age groups. Identification of SEP groups with an inequitable vulnerability to falling may guide public health practitioners and healthcare clinicians as to which populations and/or types of patients who are most in need of fall-related interventions. Further, understanding life course processes of associations between SEP and falls may inform the targeting of interventions to appropriate periods in the life course to maximise effectiveness in reducing fall-related morbidity and mortality.

## supplementary material

10.1136/bmjopen-2024-087971 online supplemental file 1

## References

[1] World Health Organization (2008). Ageing Life Course Fam Community Health WHO Glob Rep Falls Prev Older Age.

[2] Salari N, Darvishi N, Ahmadipanah M (2022). Global prevalence of falls in the older adults: a comprehensive systematic review and meta-analysis. *J Orthop Surg Res*.

[3] Montero-Odasso M, van der Velde N, Martin FC (2022). World guidelines for falls prevention and management for older adults: a global initiative. Age Ageing.

[4] Centers for Disease Control and Prevention (2023). Facts about falls | fall prevention | injury center. https://www.cdc.gov/falls/facts.html.

[5] (2021). Strategies for preventing and managing falls across the life-course.

[6] Hartholt KA, van Beeck EF, Polinder S (2011). Societal Consequences of Falls in the Older Population: Injuries, Healthcare Costs, and Long-Term Reduced Quality of Life. J Trauma.

[7] Scheffer AC, Schuurmans MJ, van Dijk N (2008). Fear of falling: measurement strategy, prevalence, risk factors and consequences among older persons. Age Ageing.

[8] Talbot LA, Musiol RJ, Witham EK (2005). Falls in young, middle-aged and older community dwelling adults: perceived cause, environmental factors and injury. BMC Public Health.

[9] Peeters G, van Schoor NM, Cooper R (2018). Should prevention of falls start earlier? Co-ordinated analyses of harmonised data on falls in middle-aged adults across four population-based cohort studies. PLoS One.

[10] Khow KSF, Visvanathan R (2017). Falls in the Aging Population. Clin Geriatr Med.

[11] Galobardes B, Shaw M, Lawlor DA (2006). Indicators of socioeconomic position (part 1). J Epidemiol Community Health.

[12] Krieger N, Williams DR, Moss NE (1997). Measuring social class in US public health research: concepts, methodologies, and guidelines. Annu Rev Public Health.

[13] Ni Y, Zhou Y, Kivimäki M (2023). Socioeconomic inequalities in physical, psychological, and cognitive multimorbidity in middle-aged and older adults in 33 countries: a cross-sectional study. Lancet Healthy Longev.

[14] Wagg E, Blyth FM, Cumming RG (2021). Socioeconomic position and healthy ageing: A systematic review of cross-sectional and longitudinal studies. Ageing Res Rev.

[15] Kwon E, Park S, Lee H (2023). Early-Life Socioeconomic Disadvantage and Health in Late Middle-Age: Importance of Heterogeneous Income Trajectories. Res Aging.

[16] Wagner C, Carmeli C, Chiolero A (2022). Life course socioeconomic conditions and multimorbidity in old age - A scoping review. Ageing Res Rev.

[17] Wagner C, Carmeli C, Jackisch J (2024). Life course epidemiology and public health. Lancet Public Health.

[18] Bloch F, Thibaud M, Dugué B (2010). Episodes of falling among elderly people: a systematic review and meta-analysis of social and demographic pre-disposing characteristics. Clinics (Sao Paulo).

[19] Todd C, Yardley L, Ben-Shlomo Y (2010). Are falls related to socio-demographic variables: a systematic review. Inj Prev.

[20] Wehner-Hewson N, Watts P, Buscombe R (2022). Racial and Ethnic Differences in Falls Among Older Adults: a Systematic Review and Meta-analysis. J Racial Ethn Health Disparities.

[21] Choi SD, Andrade EL (2022). Multiple-Level Fall Risks Associated with Socioeconomic Factors Among Community-Dwelling Older Adults: A Systematic Review. *ISER*.

[22] Bailey C, Jones D, Goodall D (2014). What is the evidence of the experience of having a fall across the life course? A qualitative synthesis. Disabil Health J.

[23] Shamseer L, Moher D, Clarke M (2015). Preferred reporting items for systematic review and meta-analysis protocols (PRISMA-P) 2015: elaboration and explanation. BMJ.

[24] Veritas Health Innovation Covidence systematic review software. www.covidence.org.

[25] van Zwieten A, Dai J, Blyth FM (2024). Overadjustment bias in systematic reviews and meta-analyses of socio-economic inequalities in health: a meta-research scoping review. Int J Epidemiol.

[26] Westreich D, Greenland S (2013). The table 2 fallacy: presenting and interpreting confounder and modifier coefficients. Am J Epidemiol.

[27] Van Kuijk SMJ, Dankers FJWM, Traverso A, Kubben P, Dumontier M, Dekker A (2019). Fundamentals of Clinical Data Science.

[28] Schooling CM, Jones HE (2018). Clarifying questions about “risk factors”: predictors versus explanation. Emerg Themes Epidemiol.

[29] van Diepen M, Ramspek CL, Jager KJ (2017). Prediction versus aetiology: common pitfalls and how to avoid them. Nephrol Dial Transplant.

[30] Hayden JA, van der Windt DA, Cartwright JL (2013). Assessing bias in studies of prognostic factors. Ann Intern Med.

[31] Hammer GP, du Prel J-B, Blettner M (2009). Avoiding bias in observational studies: part 8 in a series of articles on evaluation of scientific publications. Dtsch Arztebl Int.

[32] Bero L, Chartres N, Diong J (2018). The risk of bias in observational studies of exposures (ROBINS-E) tool: concerns arising from application to observational studies of exposures. Syst Rev.

[33] ROBINS-E Development Group (2023). Risk of bias tools - ROBINS-E tool. https://www.riskofbias.info/welcome/robins-e-tool.

[34] Schisterman EF, Cole SR, Platt RW (2009). Overadjustment Bias and Unnecessary Adjustment in Epidemiologic Studies. Epidemiology (Sunnyvale).

[35] Egger M, Davey Smith G, Schneider M (1997). Bias in meta-analysis detected by a simple, graphical test. BMJ.

[36] West SL, Gartlehner G, Mansfield AJ (2010). Comparative effectiveness review methods: clinical heterogeneity. http://www.ncbi.nlm.nih.gov/books/NBK53310/.

