# Supplementary material for: Socioeconomic position across the life course and falls among middle- and older-aged adults: protocol for a systematic review
Source: BMJ Open. 2025 Jan 21;15(1):e087971. doi: 10.1136/bmjopen-2024-087971 (PMC11784422; doi:10.1136/bmjopen-2024-087971)
Supplement: Supplementary file 2 [file bmjopen-2024-087971.reviewer_comments.pdf]

PEER REVIEW HISTORY

BMJ Open publishes all reviews undertaken for accepted manuscripts. Reviewers are asked to complete a checklist review form and are provided with free text boxes to elaborate on their assessment. These free text comments are reproduced below.

ARTICLE DETAILS

Title (Provisional)

Socioeconomic position across the life course and falls among middle- and older-aged adults: protocol for a systematic review

Authors

Smit, Frerik; Van Zwieten, Anita; Sherrington, Catherine; Zambelli, Marcia; Cullati, Stéphane; Blyth, Fiona; Khalatbari-Soltani, Saman

---

VERSION 1 - REVIEW

Reviewer 1

Name Pulkki-Brännström, Anni-Maria

Affiliation Umeå University, Department of Public Health and Clinical Medicine, Epidemiology and Global Health

Date 08-Jul-2024

COI I have no conflicting or competing interests.

This protocol is well-written and sufficiently detailed. The rationale and research questions are clearly stated and the methodology appears to be sound. I have only two minor observations.

1. The work is already very close to being completed (July 2024). The timing of the protocol is not ideal.

2. Given that the inclusion criteria are very generous in the sense that there are no restrictions regarding language, geography or date of publication, it is somewhat surprising there is no description of how heterogeneity in study contexts will be considered in the analysis phase. For example, whether participants are community-dwelling or not. Another relevant dimension could be rurality.

VERSION 1 - AUTHOR RESPONSE

Reviewer comments:

Reviewer point 1: *"This protocol is well-written and sufficiently detailed. The rationale and research questions are clearly stated and the methodology appears to be sound."*

Author response:

We thank the reviewer for these kind words about our manuscript.

Reviewer point 2: *"The work is already very close to being completed (July 2024). The timing of the protocol is not ideal."*

Author response:

As noted in our response to editor point 1, we have not yet reported the results of this review. Further, as these revisions do not include any major changes to the protocol that we submitted in April 2024, we are happy to proceed with the publication of this protocol.

Reviewer point 3: *"Given that the inclusion criteria are very generous in the sense that there are no restrictions regarding language, geography or date of publication, it is somewhat surprising there is no description of how heterogeneity in study contexts will be considered in the analysis phase. For example, whether participants are community-dwelling or not. Another relevant dimension could be rurality."*

Author response:

We thank the reviewer for noting this important aspect of the likely heterogeneity of study contexts across included studies. Given that syntheses are conducted separately by each SEP indicator and that we are incorporating life course considerations, a priori, we decided to conduct sub-group analyses by age, sex, ethnicity/race, and comorbidities if an adequate number of studies with sufficient homogeneity are found. As highlighted under the "data extraction" subheading, we planned to extract detailed data on participants' characteristics and study settings, which will be captured in our narrative syntheses.

Discussion – pages 15-16: In the limitations paragraph we have added to the discussion, we have included the following sentence to acknowledge this limitation:

- *"Similarly, given that no inclusion restrictions are placed based on study language, geography, date, and setting, considerable heterogeneity across study contexts is expected. Therefore, meta-analyses or sub-group analyses may not be feasible. However, we will discuss this heterogeneity in detail through narrative synthesis."*
